# Impact of oral health on Australian urban Aboriginal and Torres Strait Islander families: a qualitative study

**DOI:** 10.1186/s12939-019-0937-y

**Published:** 2019-02-18

**Authors:** Kaley Butten, Newell W. Johnson, Kerry K. Hall, Maree Toombs, Neil King, Kerry-Ann F. O’Grady

**Affiliations:** 1 Queensland University of Technology, Institute of Health & Biomedical Innovation, Centre for Children’s Health Research, 62 Graham Street, South Brisbane, Qld, 4101 Australia; 2 Menzies Health Institute Queensland and School of Dentistry and Oral Health, Gold Coast Campus, Griffith University, Gold Coast, Qld, 4222 Australia; 3 Dental Institute, King’s College London, London, UK; 4 Griffith University, 170 Kessels Road, Nathan Campus, Qld, 4111 Australia; 5 Rural Clinical School, The University of Queensland, 152 West St, South Toowoomba, Qld, Toowoomba, 4350 Australia; 6 Faculty of Health, Queensland University of Technology, Queensland, Victoria Park Road Kelvin Grove, Qld 4509 Australia

## Abstract

**Background:**

The oral health of a child not only impacts the physical well-being of the child, but can have quality of life implications for parents and families as they endeavour to provide care and support their child’s oral health needs. Within Australia, Aboriginal and Torres Strait Islander children are thought to experience a disproportionate burden of poor oral heath compared to non-Indigenous children. Despite the prevalence of oral health challenges, there are limited qualitative studies investigating the oral health experiences of families. The objective of the study was to explore ‘from the perspective of urban, Aboriginal and Torres Strait Islander parents and carers’ the impact child oral health has on families.

**Methods:**

Yarning circles and face-to-face interviews were used to document the experiences of (*N* = 20) parents of urban, Aboriginal and Torres Strait Islander children. Participants were recruited from an Aboriginal-owned and operated primary health clinic in northern Brisbane, Australia and through word of mouth. Information collected was transcribed and analysed thematically. Codes and themes were confirmed by the researcher and two participants.

**Results:**

The findings indicate that oral health is an important issue for urban Indigenous families and maintaining oral health to a desired standard is having emotional, physical and financial impacts. Themes identified were financial concerns, worry about the future and juggling multiple priorities, all of which were inter-related and cyclical.

**Conclusions:**

Families in this study have demonstrated that with the current policy arrangements, oral health is impacting their quality of life, contributing to stress, financial challenges and at times affecting their physical health. To address these challenges, oral health education and promotion needs a multidisciplinary approach that reaches families before children are school-aged.

## Background

The physiological impacts of poor oral health on a child are well described [1, 2]. Oral health is known to influence systemic health over the life course [3] and can be associated with significant pain and morbidity [4]. Less researched is the psychosocial impact the oral health of a child or children can have on parents and families. Psychosocial factors relate to a person’s environment and their mental, emotional and social response to their environment and experiences. Parents are typically responsible for their child’s oral health, facilitating preventative behaviours, providing care and comfort, and organising and paying for treatment when needed. Providing this support, they and their family’s lives can be impacted by a child’s oral health needs [5–8].

Within Australia, Aboriginal and Torres Strait Islander (hereafter respectfully referred to as Indigenous) children experience worse oral health than non-Indigenous children [9]. Indigenous children experience more caries, are twice as likely to be hospitalised for oral health problems and are more likely to require extraction and receive general anaesthetic than non-Indigenous children [10]. Despite the prevalence of oral health issues for Indigenous children, there has been scant research that aims to understand how oral health is impacting family life from the perspective of parents and carers [11]. In other populations, studies have demonstrated that a child’s oral health can negatively impact both their own quality of life and that of their parents [12]. Parents have reported feeling ‘bad’, ‘guilty’ and ‘upset’ [12–14], and have experienced financial impacts, attributed to decreased working hours while seeking and paying for treatment [13, 15].

Understanding how oral health is experienced by families is necessary to inform policies and programs that aim to close the gap in Indigenous oral health. This study aimed to understand, from the perspective of parents and carers of Indigenous children, how child oral health impacts on their families’ overall well-being.

## Methods

### Setting and context

This qualitative study was conducted after the completion of a prospective cohort study [16] that investigated the respiratory health and oral health of young Indigenous children in Caboolture. Caboolture is a northern suburb of Brisbane, which is the capitol city of Queensland (QLD), Australia. Between February 2013 and October 2015, the prospective cohort study used a parent/carer questionnaire to collect data on demographic, cultural, economic, and clinical factors relating to respiratory and oral health as well as conduct opportunistic oral health examinations. The full study protocol and results have been published [16–18]. Recruitment for this qualitative study commenced in August 2017 within the same Caboolture population. According to the 2016 Australian National Census, 4.8% of Caboolture’s population of 67,460 people identified as Indigenous Australians. The Indigenous population being higher than the rest of QLD (4.0%) and Australia (2.8%) [19]. Demographic data collected previously indicates that the study population is considerably economically disadvantaged; 42.7% of fathers and 85.5% of mothers were unemployed and 76.6% of households had an annual income of <$37,500 (US) [18].

Within Australia, oral health care is not included under the public health care system, Medicare [20]. Although a public dental care system exists, it is stipulated by eligibility criteria which varies by state and territory [20]. In QLD, adults must have a concession card, which is provided on application by a national social services agency such as Centrelink or the Department of Veterans’ Affairs [20]. For children and adolescents aged 2–17, a national program, The Child Dental Benefits Schedule (CDBS) provides a $1000 voucher per eligible individual every 2 years (child must be eligible for Medicare and be of a family that receives Family Tax Benefit A) which can be used in state or private dental services. Services are limited and include basic prevention and treatment options including clinical examinations, diagnostic radiography, tooth cleaning and fillings [21]. State provided oral health services in QLD for children and adolescents have traditionally been provided through the School Dental Service [22]. Over time, these services have changed in their regularity and some locations have moved into hospital and/or clinic sites. Currently, the State Health Department advises that services for children/adolescents should be provided at least once every two years [22], in accordance with Australia’s National Oral Health Plan 2015–2024 [23]. School dental services are delivered to primary schools and only children that have returned a consent form signed by a parent/carer can receive care [24]. Indigenous Australians are entitled to use Aboriginal Community Controlled Health Services (ACCHS). However, like the State services, ACCHS operate using the CDBS voucher and have options for private service [24]. These services vary by location. It is believed that the public health system only covers treatment for 20% of concession card holders [24]. Any care required outside of the CDBS limits (e.g., orthodontics), needs to be undertaken privately [21]. Private oral health services can be subsidised at the time of presentation by an individual’s private health insurance. However, within the study area, only 5% of families reported having private health insurance [18].

### Design

This study used a qualitative approach to investigate the impact of child oral health on the well-being of Indigenous families in an urban community north of Brisbane. Parents/carers were eligible if they were or had previously been the carer of an Indigenous child aged five years old or younger. Participants were recruited from a previous oral health study [16–18] and through word-of-mouth at an Aboriginal-owned and operated primary health care clinic. Both an Indigenous and non-Indigenous researcher were involved in the recruitment of participants. Participants were given the option to have both researchers present if they preferred, or if comfortable, could arrange a time with the primary researcher who is a non-Indigenous Canadian/Australian. The study protocol was explained verbally and through a written plain language statement that was provided to potential participants. Signed consent was obtained. Participants were offered the choice to meet at the health clinic or at a location comfortable for both them and the researchers. Recruitment continued until saturation with respect to information shared in the discussions.

The study followed a social constructionist perspective [25], whereby knowledge is socially created by the participants and researcher and yarning methods were used for data collection: sharing information verbally through a relaxed discussion is an accepted method of exchanging knowledge within Aboriginal culture and is commonly described as yarning [26]. The yarns were led by a non-Indigenous researcher with the assistance of an Indigenous researcher. The researchers shared information about the previous quantitative study [18] and described the qualitative study as an opportunity to share their perspective as opposed to answering questions on a paper survey. As part of the yarning process, and to establish rapport with participants, the non-Indigenous researcher shared her personal experiences with oral health including her experience as mother to two boys. The yarns began with a starting question such as, “can you tell me about what oral health is like for your family?” and were given the opportunity to follow the narrative of the participant. Semi-structured questions were available if needed, for example, “what are some of the things you find easy or hard when it comes to oral health for your kids?”. All sessions were recorded with a digital audio recorder (Philips Digital Voice Tracer LFH662, Korea).

### Analyses

The information gathered was transcribed by two authors (KB and KH). The data were entered into the software package NVivo 11.4 and coded thematically by the researcher (KB). Codes established from the data were then printed out onto paper. The researcher returned to two participants who had expressed interest in contributing to the study process for their input into coding and theming. All other participants were offered the opportunity to participate, but declined or were unreachable upon 3 contact attempts. This method of analysis was both verbal and tactile. The words/phrases were placed on pieces of paper and the participants and researcher could move them into groups, as well as add anything they thought was missing. This element of analysis was important to countering the subconscious bias and ethnocentrism that can occur with non-Indigenous participation in research [27]. It also aimed to honour the collaborative process and the notion that the participants are the experts on their own lives. The final themes were subsequently cross-referenced in NVivo. This study was approved by the Queensland Children’s Hospital and Health Service Human Research Ethics Committee (HREC/17/QCRH/94).

## Results

Participants (*N* = 20) were all mothers with the exception of one carer who was an aunt. The predominance of mothers in the sample is likely reflective of both the recruitment process and the natural division of care within the study population. Although fathers and other carers were invited to the study, the majority of participants were recruited from the prior prospective cohort study whereby participants were recruited opportunistically from a health clinic while attending appointments with their children. In this case, mothers were the predominant carer in attendance with their children. There were 2 group sessions consisting of 3 and 5 participants, and 12 individual yarns. Both researchers were present for the group sessions and for 3 of the interviews. The other 9 interviews were conducted by the non-Indigenous researcher independently. No difference in data, in terms of collection or results, was perceived by the non-Indigenous researcher when conducting the interviews. The duration of the yarns ranged between 35 min and 2 h. Many participants also had experience caring for children other than their own, either through family responsibilities and/or in a professional capacity working in day care or education. When yarning, participants often drew upon these experiences and shared their knowledge and perception of how other Indigenous families were impacted by oral health as well as themselves. Although no specific demographic details were collected in this study, the participants were recruited from a predominantly low socio-economic population; 76.6% of families from the same study population had an annual household income of <$37,500 (US) [18].

Overall, mothers explained that when their children were very young, they felt efficacious in their approach to oral health and it was something that was at times stressful, but manageable. All mothers were aware that oral health was relevant to their young children and had introduced oral health behaviours such as brushing with toothpaste and limiting sugar. However, as children aged, mothers expressed how oral health had a greater impact on their family lives. Although some mothers accepted caries as a normal part of childhood, prevention and acute issues, such as the need for orthodontic treatment, were taken seriously. The impact of dealing with these issues was multifaceted, experienced both emotionally and physically as well as affecting finances and personal resources.

**Fig. 1:**
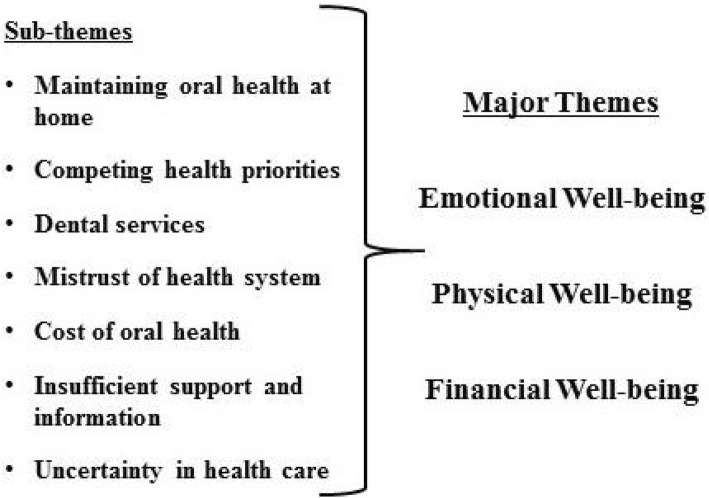
Relationship of themes

There were 3 overarching themes identified: emotional well-being, physical well-being, and financial well-being. These themes overlapped and were interrelated and were present within the sub-themes as shown in Fig. 1. In this study, emotional well-being refers to the mental load and emotional and personal resources required to maintain the participants’ desired level of oral health for their child. Maintaining oral health at home, engaging health services and competing health priorities all impacted emotional well-being; as did considering whether they were doing enough for their children’s health, whether services were available and appropriate, whether they could afford treatment and a concern for their family’s future oral health. Physical well-being largely refers to children’s experiences as described by their parents; with children experiencing direct physical impacts of oral health problems or indirectly, because of related circumstances. Impacts to financial well-being were experienced at a number of different levels, but were most severe when children had oral health needs that required intervention and as children aged. The influence of age and financial challenges permeated the discussion, as many participants related their own oral health experiences as they aged and their concerns for their growing children. The results have been organised under each sub-theme as the sub-themes often correspond to more than one major theme.

### Maintaining oral health at home

All mothers were aware of the importance of oral health and strived to have their children brushing at least once a day. Upholding oral health behaviours at home was experienced differently between mothers. Some mothers described their children’s relationship to oral health habits as being good and consistent, and felt that they were lucky to have kids that practised desirable oral health behaviours. Other mothers described implementing and upholding oral health habits as far more challenging and contributing to stress for themselves and children.

*“It causes a lot of stress in our house. Mainly for the younger two and for my second born son and because he doesn’t like doing it or he forgets.” (M1 - interview).*

Mothers offered different explanations for the challenges. Children’s age (younger and older children presented different challenges), and a child’s personal perception of oral health and family structure were primarily raised as impediments to oral health routines. There was a sense of exasperation around upholding oral health habits at home.

*“I was a single parent right from the time he was born and had that shared care stuff, so that’s the difficulty with which I see now so much with the parents that I see, is that your role and parents having such conflict in their homes and then pressure of working and things whatever, so those general routines fall through the cracks or they have a routine but they don’t have it in the other home and they have no control of implementing that.” (M2 - group)*

### Competing health priorities

Children’s mental and physical health needs were discussed in relation to oral health. Mothers explained how other health needs impacted on their child’s oral health habits, particularly where medication was needed. One mother explained how her son’s autism was a barrier to his oral health because of his vehement opposition to routine tooth brushing. The mother explained that trying to support him with his oral health on a daily basis was very stressful. Other mothers described how chronic health issues which required medications directly impacted on their child’s teeth and the efforts they had to go to support their child’s oral health were taxing mentally and physically. Certain medications can increase the risk of caries by lowering the pH of the mouth and/or exposing the teeth to sugars [28]. Parents are often advised to either rinse their child’s mouth or brush their teeth after each dose.

*“Having a health problem changes everything... it impacts on me at midnight and at 4 in the morning because I wanna make sure her teeth are happy and healthy too.” (M3 - interview).*

Many participants with children with other health needs explained their situation as one in which oral health was just one of the many things that they were juggling. Oral health was not always the top priority. There were also perceptions that when children were young, missing preventative care was not as important as when children were older and had their permanent teeth.

*“She’s got lung problems, she’s anorexic, which is also probably destroying her teeth, umm she’s got mental health issues as well. Umm the next child down, they think she has possible EDS (Ehlers Danlos Syndrome) so there’s a whole range of specialists there we need to start seeing, and orthopaedics. Rheumatoid specialists because of the pains she is in. You know there’s just all those sort of things. My youngest has cardiac specialists, she has a hole in her heart and global developmental delay.”(M4 - interview).*

### Dental services

Few mothers took their pre-school children to the dentist, but most participants indicated that school dental vans were well utilised for older children. Mothers considered the school service, coupled with the CDBS [21], beneficial because it provided an opportunity to see the dentist before there was a problem. Many participants suggested that they themselves would be unlikely to seek preventative treatment, mostly because of the cost.

*“At the school they are so wonderful. My kids are scared of dentist and they are wonderful and he wants to go there all the time, every time his teeth hurt he wants to go there, so it’s really good, so we are quite impressed with the way they handle the children there. It’s about the children’s opinion and what they want and they make them feel safe and secure.” (M5 - interview).*

Other mothers suggested that the current system had problems and the services available were not to a high standard, not regular enough and not practical for parents.

*“When Medicare took over and started issuing those vouchers, parents had to attend the dentists, the school dental van with the children, which a made it really hard, because if they can’t get you in, you have to take time off work. I mean if its big procedure you want to be there, but if it’s their standard check-up it’s no big deal, they are big kids so to speak, but that impacted how often kids were seeing the dentist.” (M6 - interview).*

*“No, they went once and they just didn’t do a proper job, you know they didn’t clean or do what I know they are supposed to do.” (M4 - interview).*

### Mistrust of health system

Mistrust and apprehension towards the oral health services provided by the schools was raised, as well as shame and fear regarding the motives of the oral health services. These feelings were transgenerational and based on personal experiences. For example, one participant had grown up in a rural location and one night she tripped and broke her tooth. Her mother brought her to the ‘city’ to have it fixed and the dentist reported her family to social services for suspicions of abuse/neglect which was proven unfounded.

*“With some of the Indigenous families, because it was then a government initiative, the dentist was a government initiative but now Medicare required a signature and signing a government form when you don’t know the terms and conditions does bring about a bit of fear like what are you signing, then there’s the other thing, the fear of what my mum went through like when they questioned how I broke my tooth, umm so the shame and the wariness as to what they are going to do, are they going to take their kids away because of bad teeth.” (M6 - interview).*

A similar sentiment of mistrust and apprehension was expressed in regards to private dental services, particularly in relation to fees.

*With the dental were you go to the hospital and that, they don’t offer root canals and things like that, you got the basics. If it can’t be filled well then it’s pulled out. You don’t have a choice when you don’t have money to go to the dentist. At the dental hospitals and clinics it’s mainly pulled. They don’t do a lot. (M12 - group).*

*“The other option is taking them to a private dentist - well they just take advantage. Unfortunately that’s all they do, is do a clean and charge you nearly $200 and tell you that will need to pay money if you need to get anything else done.” (M6 - interview).*

### Cost of oral health

Some participants felt that current oral health services were insufficient for their child’s needs. Those with children that had oral health needs outside the school’s dental service capacity explained that their child’s oral health requirements exceeded the CDBS. Thus, parents had to go into debt to afford interventions putting costs on credit cards and/or using payment plans. Others offered that to make ends meet, their children had to forgo activities, giving up a sport or an extra-curricular activity.

*“It cost us eight grand and we had to go into debt to get that but you do it for your kids there is no reason not to and um, but we are slowly getting back on our feet and that type of thing, but you do it for your kids.” (M7 - interview).*

For some, finances were major impediments and their children missed out on treatment because of the cost. These situations were upsetting to mothers and they expressed their helplessness in not being able to solve their child’s health problems despite their engagement with the health system and attention to the problem. There was also an acknowledgment that the overall health and well-being of their children were being negatively affected by their oral health needs and lack of care by the health system.

*“We have always had extra teeth that like grow out of the bottom. I had like two pair that were removed when I was younger because of the overcrowding in the mouth and everything. But my sons are that bad that he actually has to have surgery for his, and it’s more for his jaw than actually his teeth… that’s something I can’t afford unfortunately” (M8 - group).*

### Insufficient support and information

Participants felt there was a lack of support when it came to referral pathways and options to address orthodontic and acute oral health needs. Mothers whose children required more complex care felt that they were left to navigate private treatments on their own and that there were limited options for those not financially privileged. Others were unsure of who to contact and often didn’t have the time and resources to find other options. One participant in particular described how, coupled with her daughter’s comorbidities, her oral health issues were contributing to her malnourishment. The mother had been in contact with approximately 16 private dentists and the dental universities to try and find an affordable option for her child’s treatment to no avail.

*“And like even for some of the dental places, I have called umm, they said they will do a payment plan but they want at least 50% up front and then they’ll do the payment plan and I don’t know if you pay them 50% then they’ll do the braces or take the 50% and then do the payment plan. You know no one has explained even how that works. You know that is going through a private dentist and I think that is $7500 for just stock standard braces, that is not including any after care, which will be through the roof. And I’ve got two that need braces.” (M4 - interview).*

### Uncertainty in health care

There was a consensus of concern for the future of their children’s oral health amongst participants. Informed by their own experiences, parents were very worried for their children as the children grew up. They felt that along with potential oral health needs, such as braces and wisdom teeth removal, oral health seemed to decline with age. The majority of participants had had their own oral health problems, had experienced tooth loss, and were unable to afford corrective treatment for themselves. A few participants shared that they felt poor oral health was inevitable, irrespective of good oral hygiene. They attributed lifestyle factors such as smoking and other comorbidities such as diabetes to the decline in oral health with age.

*“I stress big time about if she will ever need braces. I know it is probably just part of my anxiety, worrying about things that haven’t happened yet, but yeah I do worry that I can’t afford everything. I get that worried that I start to cry about it. You know, the financial situation. I’ve been financially strapped for a long time, but I’m used to it, but then I start thinking about all the things they miss out on, instead of it just being me, it’s just us.” (M10 - interview).*

## Discussion

In this study we aimed to understand from the perspective of urban Indigenous parents and carers how child oral health was impacting on their family life. According to national statistics [9], Indigenous Australians of all ages experience worse oral health outcomes than non-Indigenous Australians, yet there are few data that explores oral health from an Indigenous Australian personal perspective [9]. We found that oral health was an important issue for urban Indigenous families and maintaining oral health to a desired standard was impacting mothers and their families. These impacts fell under three major themes: emotional well-being, physical well-being and financial well-being. Sub-themes identified were inter-related and did not necessarily fit under one major theme, but were shared across themes. The interconnectedness of the findings suggest that the impact of child oral health as reported by mothers is complex and is not limited to the individual child or parent, but experienced by the whole family as well.

Although oral health research has typically had an individual focus, consideration has also been given to a person’s environment, and in child oral health, the relationship dynamic with parents. In our study, mothers had different experiences of maintaining oral health at home, some positive and others stressful. As in other studies [29], mothers in our study reported child un-cooperativeness as a common barrier to maintaining oral health. Research has suggested that parents who have reported positive oral health behaviours for their children have higher levels of oral health knowledge and favourable oral health behaviours themselves [30]. Increasing the self-efficacy of parents could potentially ameliorate some of the challenges that parents face when supporting child oral health. However, consideration and support would need to be given to parents, particularly mothers’, individual circumstances. As is typical for many families, mothers perform the majority of childcare duties. This study and others [31] have found the same is true for Indigenous families. Data from the Longitudinal Study of Indigenous Children indicates that while fathers do participate in supporting oral health, 28% of fathers reportedly helped their child brush their teeth ‘most days’, fathers are generally less likely to be involved in personal care activities [31]. Participants in our study noted the challenges for themselves, but also in the context of shared care and coordinating oral health routines between separated parents. Single-parent families have been found to be an associated risk-indicator for child dental caries [32], which could be indicative of the additional family stress that parents are experiencing, as well as the reduced personal resources related to separation [32]. More research is needed to understand the complexities of the family dynamic on oral health and how parents can be supported in sharing the responsibilities of oral health in all family situations.

The ubiquity of oral health maintenance has potentially contributed to the mindset that achieving ‘good oral health’ is the same for everyone and simply involves regular brushing, flossing and limiting sugar. Little thought is given to the competing health priorities of individuals, let alone families. While it was positive to hear that parents are recognising the cariogenic potential of medications, it is important to understand the knock-on effects for families and how they are coping with these additional requirements. Like our study, other studies [33] have found that parents of children with additional health needs report increased stress and worry, impacts to sleep, and parent-child conflict [33]. In an oral health context, one way of coping with these additional challenges is the rearranging of health priorities and oral health potentially being overlooked. The perception of oral health as being a non-essential health priority has been reported by researchers in other studies [34]. Moreover, the age of the child also seems to affect this perception, with baby-teeth perceived to be less important than permanent teeth [34]. This notion unfortunately can have ramifications for oral health later on and contribute to additional health challenges for children and parents. There has been considerable advocacy for the inclusion of oral health promotion into primary care services to increase parent awareness of oral health requirements related to overall health [35]. However, families in our study demonstrate that they are cognisant of oral health, but are struggling regardless. Measures to support families with complex health situations need to be targeted and provide practical strategies that parents can implement.

The cost of oral health care in Australia was a predominant concern for participants. Although the school services and CDBS were generally well-received, they were often insufficient. Families in this study shared the sacrifices they and their children had made to maintain their health. When a monetary sacrifice could not be made, it was a child’s health and a parent’s well-being that suffered. Participants shared how, in some circumstances, their children had to go without care, all because of the location of their health issue, their mouth. According to an Australian National Health Survey, 10.7% of children aged 5–14 had avoided or delayed oral health treatment because of the cost; likewise for 44.9% of adults aged 25–44 [36]. These sorts of financial/health dilemmas are often not associated with publically funded health care and yet, oral health remains a barometer for wealth in many countries including Australia [35].

Families indicated that they were aware of these disparities in care and this awareness contributed to anxiety and concern, even before problems or health needs had arisen. Mothers of young children forecast their struggles, anticipating the need for interventions and their potential inability to meet the costs. Tooth loss was a particular concern as it was a common problem for the participants themselves. In Australia, there is a near linear relationship between the number of missing teeth and annual income [37], and the same can be found in other countries [38]. The CDBS and school services were reportedly well-used by our study participants, but most indicated that as adults they were unlikely to go to the dentist, citing cost as the primary reason for their avoidance. Others have reported similar findings [39] and larger studies have demonstrated that those with lower incomes are the least likely to attend the dentist for preventative care [20]. These financial barriers have wider implications than just immediate care. Primary oral health care services provide an opportunity for health promotion, patient education and building on self-efficacy [40], which may support oral health behaviours that reduce the risk for poor oral and general health. Increased self-efficacy has been demonstrated to have positive implications for (oral) health for all individuals regardless of their socio-economic status [41].

Again, the “siloing” of oral health from primary care is a likely barrier to education and information for low socio-economic families [42]. Parents in our study indicated that there was little guidance when it came to their family’s oral health needs and services. In a similar study, Indigenous mothers and carers from Western Australia expressed their desire for more education and clear, practical reasons and ways to change behaviours in regards to oral health care [11]. According to a 2014 review [43] of paediatric oral health information within Australia, there are considerable informative resources available. However, the readability of the materials is low and those that are reader-friendly have little useful information [43]. There also appeared to be very limited culturally relevant material [43]. Effective and respectful communication with Indigenous people is something by which Western health systems are commonly challenged [44]. Historical racism and paternalism have fostered transgenerational inequities in care and a mistrust that continues to impact on the health of Indigenous people [44]. As some participants shared, there is a wariness of both the public and private services; and participants do not feel supported throughout their life course. Lack of support and mistrust of health services is commonly reported by Australia’s Indigenous population [45]. These findings are important for oral health professionals who are interested in reducing the disparities in oral health, because although Australia’s current oral health policy is focused on children, parents are known to have considerable influence on their child’s oral health [46]. More research is needed to understand how best to engage parents within marginalised populations and what information would be most helpful to them and in what format.

As has been highlighted, the impact of oral health on urban Indigenous families can be attributed to a complex interplay of environmental and socio-ecological factors. Emotional, physical and financial well-being do not stand independently of each other, but are interconnected and largely co-dependent. At present, the cost of oral health for adults in Australia has arguably influenced the uptake of oral health services for families, which in turn limits the opportunities for support and education across the life course. Families are trying to manage with limited personal and professional resources, which is affecting well-being. Addressing the impact of oral health on families and supporting well-being will require a multi-faceted approach, one that considers both the needs of the child, but also the capacity of the parent and family to support the desired standard of oral health.

### Limitations

Our study is not without limitations. Although the sample of this study was small, participants consistently shared similar experiences, suggesting that saturation was met for the overarching themes. However, as the participants were drawn from one location, the findings may not be generalisable to other areas of Australia. Despite an Indigenous researcher being present for many of the yarnings, the primary researcher is a non-Indigenous Australian. This may have influenced the comfortability of the participants in sharing. However, the enthusiasm and length of the yarns suggest that participants felt at ease with both of the researchers.

## Conclusion

Families in this study have demonstrated that with the current policy arrangements, oral health is affecting their well-being, contributing to stress, financial challenges and at times affecting their physical health. To address these challenges, we recommend a multi-level and multidisciplinary approach to oral health promotion and education. Oral health promotion can no longer only be the domain of dentists, but must be embedded into the routine practices of those that work closely with urban Indigenous families on a more regular basis. The inclusion of oral health education in midwifery, nursing and medicine programs would be a good a start. Health professionals need to feel confident in oral health and in providing patients with information and pathways to support their oral health as they would any other health issue. While school dental programs are well utilised, they are potentially too late for parents who need support in fostering desirable oral health behaviours for their children when dentition commences. We recommend that oral health information and support gets to parents earlier. The inclusion of oral health promotion and screening during pregnancy is certainly beneficial, but it must be acknowledged that this approach often singles out mothers and a whole of family approach is needed. By sharing the responsibility of oral health advocacy across health disciplines, a dialogue can open up and potentially breech some of the barriers expressed by participants in our study and others.
